# Supplementary figures and images for: Repeated dermal application of the common preservative methylisothiazolinone triggers local inflammation, T cell influx, and prolonged mast cell-dependent tactile sensitivity in mice
Source: PLoS One. 2020 Oct 26;15(10):e0241218. doi: 10.1371/journal.pone.0241218 (PMC7588120; doi:10.1371/journal.pone.0241218)

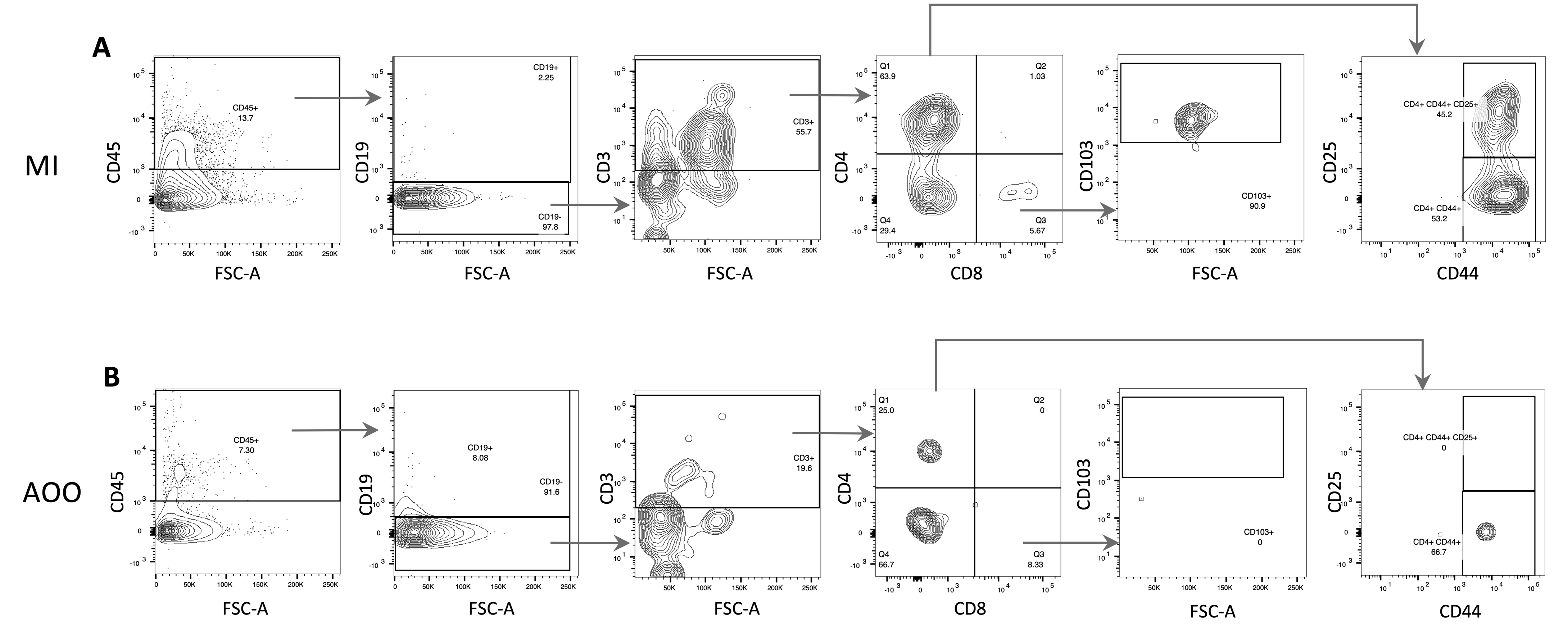

Supplement: S1 Fig — (A-B) Representative flow plots showing contour plots with 5% outliers T lymphocyte gating strategy in MI-challenged (A) and AOO-treated (B) mice. CD3+ populations were gated out of CD45+CD19- cells first. CD8+ and CD4+ were gated out of the CD3+ gate. Out of CD4+ cells CD44 and CD25 was evaluated and out of the CD8+ cells CD103 was analyzed. Results were collected on a BD Fortessa and all analysis was done using FlowJo software. (TIF) [file pone.0241218.s001.tif]

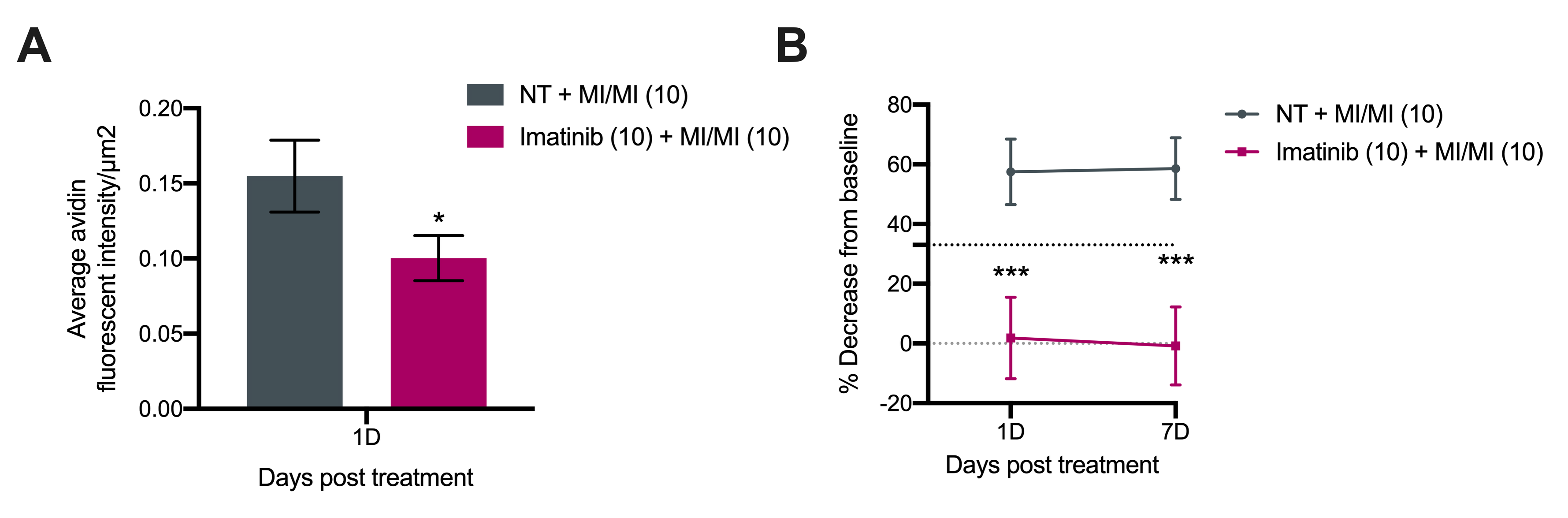

Supplement: S2 Fig — For preventative imatinib treatment, mice were treated with 100 μL of 30 mg/kg imatinib dissolved in 0.9% saline injected intraperitoneally 30 minutes before each of 10 daily MI challenges. (A) Density of avidin+ mast cells in 10 μm labiar skin cryo-sections from sensitized mice receiving NT or preventatively treated with imatinib and challenged 10 times daily with MI. Results displayed as mean ± SEM; n = 6/treatment group. Significance with respect to control group, * p<0.05. (B) Tactile sensitivity in sensitized mice preventatively treated with imatinib or NT and then 10 daily MI challenges. Results reported as mean ± SEM of the percent decrease from baseline in the withdrawal threshold for each treatment group, n = 18/treatment group. Black dotted line denotes 33% decrease from baseline and grey dotted line marks 0% decrease. Significance with respect to control group, *** p<0.001. (TIF) [file pone.0241218.s002.tif]
