# Supplementary material for: Repeated dermal application of the common preservative methylisothiazolinone triggers local inflammation, T cell influx, and prolonged mast cell-dependent tactile sensitivity in mice
Source: PLoS One. 2020 Oct 26;15(10):e0241218. doi: 10.1371/journal.pone.0241218 (PMC7588120; doi:10.1371/journal.pone.0241218)
Supplement: S1 Table — Labiar withdrawal thresholds of MI sensitized AOO- and MI-challenged mice (top) or MI-sensitized and challenged NT or therapeutic imatinib treated mice (bottom). Thresholds presented in grams as mean ± SD in mice at baseline prior to sensitization and at predetermined timepoints after challenge (n = 18). Percent change in withdrawal threshold from baseline is depicted in Fig 4 for MI/AOO (10) and MI/MI (10) mice. Withdrawal thresholds for therapeutic imatinib (MI/MI (10) + Imatinib (6)) or NT (MI/MI (10) + NT) mice are shown in Fig 5. (DOCX) [file pone.0241218.s003.docx]

| **Treatment** | **Baseline** | **1D** | **28D** | **42D** | **56D** | **70D** |
| --- | --- | --- | --- | --- | --- | --- |
| MI/AOO (10) | 0.88 ± 0.19 | 0.88 ± 0.39 | 0.75 ± 0.23 | 0.68 ± 0.26 | 0.86 ± 0.24 | 0.92 ± 0.36 |
| MI/MI (10) | 0.88 ± 0.20 | 0.38 ± 0.16 | 0.34 ± 0.12 | 0.36 ± 0.13 | 0.33 ± 0.11 | 0.42 ± 0.22 |
| **Treatment** | **Baseline** | **1D** | **7D** | **14D** | **21D** | **28D** |
| MI/MI (10) + NT (6) | 0.94 ± 0.12 | 0.49 ± 0.14 | 0.50 ± 0.09 | 0.46 ± 0.15 | 0.42 ± 0.10 | 0.44 ± 0.13 |
| MI/MI (10) + Imatinib (6) | 0.95 ± 0.15 | 1.00 ± 0.21 | 1.06 ± 0.06 | 0.91 ± 0.18 | 0.91 ± 0.15 | 0.90 ± 0.12 |
