# Supplementary material for: Repeated dermal application of the common preservative methylisothiazolinone triggers local inflammation, T cell influx, and prolonged mast cell-dependent tactile sensitivity in mice
Source: PLoS One. 2020 Oct 26;15(10):e0241218. doi: 10.1371/journal.pone.0241218 (PMC7588120; doi:10.1371/journal.pone.0241218)
Supplement: Attachment — Submitted filename: Response to Reviewers.docx [file pone.0241218.s004.docx]

**Response to Reviewer 1 Comments**

Point 1: This study lacks novelty and most of the results have been already previously published by this group.

We thank the reviewer for their careful and comprehensive reading of the manuscript. We acknowledge the reviewer’s critique disputing the novelty of this study given we have recently published similar findings via intravaginal application of MI. However, we respectfully submit that given the completely different route of MI application, this study 1) validates findings that link occupational exposures to vulvodynia with the skin being a more probable exposure site and 2) demonstrates the capacity of skin responses to MI to be dramatically more pronounced than that within vaginal mucosal tissues. It is well known that route of administration can dramatically affect allergic responses^1^. However, this has yet to be shown for MI and in particular, to our knowledge it has not been shown to be linked to intensity and duration of MI-induced pain. Furthermore, although there is a growing body of work demonstrating rapid increasing prevalence of MI skin allergy particularly in the US and Europe, published work that characterize immune dynamics of MI skin allergy are minimal. We provide a framework looking at numerous, likely important immune infiltrate at various timepoints during MI allergy which are key first steps to begin to understand immune responses to this preservative, all while linking it to development of painful responses. We have thoroughly revised both our Introduction and Discussion sections to clarify and emphasize these points.

Point 2: CD44 +CD25+ CD4 T cells as well CD44+ CD8 T cells do not represent T cell subtypes but rather activation states of the T cells. It is important to delineate between T cell subtypes and activation state.

The reviewer raised an excellent point regarding the use of the word subtypes for activated T cells. We thank them for catching this error and have removed “subtypes” and replaced with “activation states.”

**Response to Editor Comments**

Point 1: Sections of the introduction are extremely similar/verbatim to a recent publication from the group.

The editor raises an important point regarding extreme similarities between the presented introduction and a previously published introduction from our group. We thank the editor for pointing this out to us and have reframed and revised the introduction thoroughly.

2. In the mechanical hypersensitivity assays shown - all the results are shown after normalization (percent decrease etc). It is difficult to appreciate the relevance of the decreases without seeing the absolute values - perhaps a table can be shown in supplementary data to complement the normalized data shown in figures.

The editor raises an excellent point regarding the need for more transparency of the tactile sensitivity when presented as relative values. We have added a supplementary table presenting average withdrawal thresholds with standard deviation for each treatment group and timepoint to make these differences clearer.

3. The increases in NGF and cadm1 in the spinal cord is interesting, but its direct relevance to the current study is limited and therefore its inclusion in the supplementary data needs to be reconsidered.

We acknowledge the editors concern about including *NGF* and *cadm1* spinal cord expression in the supplementary data. While we had originally included it to further contextualize the increase in CGRP^+^ nerves as well as increased painful responses at the MI exposure site, we understand that the scope of these findings go beyond the focus of the current study and are not extensive enough to contribute substantially to the manuscript. We thank the editor for this comment and have removed this supplementary figure from the manuscript.
